# Metastatic organ count stratifies survival in immunotherapy-treated metastatic colorectal cancer: a retrospective cohort study

**DOI:** 10.3389/fimmu.2025.1679041

**Published:** 2025-12-09

**Authors:** Hai-Yan Fu, Yi-Yang Zhang, Yan Wang, Jin-Shan Huang, Jia-Hao Tian, Na Wang, Kun-Hao Bai, Zhi-Qiang Wang

**Affiliations:** 1 State Key Laboratory of Oncology in South China, Guangdong Provincial Clinical Research Center for Cancer, Sun Yat-Sen University Cancer Center, Guangzhou, China; 2 Department of Medical Oncology, Sun Yat-Sen University Cancer Center, Guangzhou, China; 3 Department of Endoscopy, Sun Yat-Sen University Cancer Center, Guangzhou, China; 4 Department of Anesthesiology, Sun Yat-Sen University Cancer Center, Guangzhou, China

**Keywords:** metastatic organ count, metastatic colorectal cancer, immunotherapy, prognosis, lung metastasis

## Abstract

**Background:**

Immunotherapy for metastatic colorectal cancer (mCRC) leads to varying patient outcomes owing to the heterogeneity of metastatic organs. However, whether the metastatic organ count influences prognosis in mCRC patients receiving immunotherapy remains unclear. Therefore, this retrospective study aimed to investigate this issue to derive a clear conclusion to facilitate clinical treatment.

**Methods:**

This retrospective cohort study included 208 patients with mCRC receiving immunotherapy at the Sun Yat-sen University Cancer Center. Cox regression analysis was conducted to determine variables independently associated with immunotherapy response and progression-free survival (PFS). The Kaplan–Meier curve analysis and log-rank test were used to evaluate PFS across varying metastatic organ counts. Subsequently, a nomogram was constructed for PFS prediction.

**Results:**

Patients with varying metastatic organ counts exhibited significantly different objective response rates (ORRs) (1 to ≥4 organs: 49.3%, 30.0%, 19.6%, 5.9%, respectively, *P* < 0.001). Multivariable Cox analysis identified low tumor mutational burden (hazard ratio [HR]=2.49, 95% confidence interval [CI]: 1.60–3.88, *P<*0.001), second-or-higher-line immunotherapy (HR = 2.83, 95% CI: 1.81–4.44, *P<*0.001), and high metastatic organ counts (3 organs: HR = 2.21, 95% CI: 1.40–3.51, *P=*0.001; ≥4 organs: HR = 3.85, 95% CI: 2.06–7.22, *P<*0.001) as independent factors associated with low PFS time. Additionally, patients with lung metastasis had a markedly lower ORR (14.6%) than did others (distant lymph node [30.7%], liver [30.3%], peritoneum [29.5%]; *P<*0.001).

**Conclusions:**

Increased metastatic organ count in patients with mCRC was associated with decreased immunotherapy ORR and worse prognosis, with a particularly pronounced effect observed in patients with lung metastasis.

## 1 Introduction

Colorectal cancer (CRC) is the third most prevalent malignancy worldwide and the second leading cause of mortality, with approximately 1.9 million new cases and 935,000 deaths reported in 2020 (1). Approximately 20% of patients with CRC have distant metastasis at the time of diagnosis (2). Immunotherapy has revolutionized the treatment landscape for various solid tumors, demonstrating remarkable efficacy, particularly in patients with metastatic colorectal cancer (mCRC) with deficient mismatch repair (dMMR)/microsatellite instability high (MSI-H) (3) or elevated tumor mutational burden (TMB) (4). The success of immunotherapy in this subset of patients has prompted the exploration of combination therapies for patients with mCRC with proficient MMR (pMMR)/microsatellite stability (MSS)/microsatellite instability low (MSI-L) profiles or those with low TMB levels, in an effort to enhance therapeutic outcomes.

Our review of the literature revealed that, when it comes to the efficacy of immunotherapy for various types of tumors, beyond TMB and MSI status, the condition of metastatic organs also significantly influences the effectiveness of immunotherapy. Su et al. (5) reported that, in patients with HER2+ breast cancer receiving immunotherapy, brain metastases can mimic the anti-inflammatory mechanisms of the central nervous system to evade antitumor immunity. Similarly, Conway et al. (6) reported that liver and brain melanoma metastases exhibit significantly reduced immune infiltration characterized by lower T-cell infiltration and macrophage density, potentially diminishing the response rates to immunotherapy in patients with such metastases. The heterogeneity of metastatic organs results in diverse tumor microenvironments, marked by gene profile alterations and organ-specific immune cell infiltration (7). This heterogeneity may account for varied responses to immunotherapy, suggesting the existence of organ-specific therapeutic efficacies.

Therefore, we hypothesize that an increase in the number of organs to which mCRC has spread correlates with a greater complexity and diversity of organ-specific immune escape microenvironments, ultimately resulting in reduced efficacy of immunotherapy.

In this study, we aimed to investigate independent prognostic risk factors associated with immunotherapy in patients with mCRC. Our study was designed to evaluate the oncological effectiveness of immunotherapy in mCRC, with a particular emphasis on metastatic organ count as a potential predictor of treatment response rates.

## 2 Patients and methods

### 2.1 Patients

We conducted a retrospective cohort analysis of electronic health records to identify patients diagnosed with mCRC who received at least two rounds of immunotherapy between January 2019 and January 2024 at the Sun Yat-sen University Cancer Center. The specific immunotherapy agent administered, dosages, and treatment schedules were determined by clinicians in adherence to established guidelines. We excluded patients who had prior exposure to immunotherapy or had missing crucial baseline radiographic data. A comprehensive overview of the patients’ demographic and clinical characteristics is presented in **Table 1**.

**Table 1:** Clinical characteristics and treatment response of patients with mCRC receiving immunotherapy.

Variables	Total	ORR (CR + PR), n (%)		*P* value
		Yes	No	
Age, years				0.317
≤ 45	58	22 (37.9)	36 (62.1)	
> 45	150	46 (30.7)	104 (69.3)	
Sex				0.509
Male	132	41 (31.1)	91 (68.9)	
Female	76	27 (35.5)	49 (64.5)	
BMI				0.052
≤ 23.32	124	47 (37.9)	77 (62.1)	
> 23.32	84	21 (25.0)	63 (75.0)	
Lynch syndrome				0.061^a^
Yes	9	6 (66.7)	3 (33.3)	
No	199	62 (31.2)	137 (68.8)	
ECOG PS				0.007
0	129	51 (39.5)	78 (60.5)	
≥ 1	79	17 (21.5)	62 (78.5)	
Smoker				0.279
Yes	46	12 (26.1)	34 (73.9)	
No	162	56 (34.6)	106 (65.4)	
Location of primary tumor				0.045^a^
Left	140	38 (27.1)	102 (72.9)	
Right	62	27 (43.5)	35 (56.5)	
Both	6	3 (50.0)	3 (50.0)	
Metastatic organ count				0.000
1	75	37 (49.3)	38 (50.7)	
2	70	21 (30.0)	49 (70.0)	
3	46	9 (19.6)	37 (80.4)	
≥ 4	17	1 (5.9)	16 (94.1)	
Neutrophil-lymphocyte ratio (NLR)				0.172
≤ 3.2	127	37 (29.1)	90 (70.9)	
> 3.2	81	31 (38.3)	50 (61.7)	
Adverse events				0.280
Yes	139	42 (30.2)	97 (69.8)	
No	69	26 (37.7)	43 (62.3)	
Microsatellite status				0.000
MSS/MSI-L	169	43 (25.4)	126 (74.6)	
MSI-H	39	25 (64.1)	14 (35.9)	
Tumor mutation burden (Mb)				0.000
< 10	148	35 (23.6)	113 (76.4)	
≥ 10	60	33 (55.0)	27 (45.0)	
Line of immunotherapy				0.000
First-line	61	47 (77.0)	14 (23.0)	
Second-or-higher-line	147	21 (14.3)	126 (85.7)	
Immunotherapy regimen				0.009
Solo therapy	30	16 (53.3)	14 (46.7)	
Combination therapy	178	52 (29.2)	126 (70.8)	
Total	208	68 (32.7)	140 (67.3)	

a Fisher’s exact test. Variables are expressed as n (%).

BMI, body mass index; CR, complete response; ECOG PS, Eastern Cooperative Oncology Group Performance Status; mCRC, metastatic colorectal cancer; MSI-H, microsatellite instability-high; MSI-L, microsatellite instability-low; MSS, microsatellite stable; ORR, objective response rate; PR, partial response.

This study was approved by the Institutional Review Board of the Sun Yat-sen University Cancer Center (SL-B2023-128-01). Given the retrospective nature of this study, the Institutional Review Board waived the requirement for written informed consent.

### 2.2 Data collection

Clinical information including age, sex, body mass index (BMI), Eastern Cooperative Oncology Group performance status (ECOG PS), smoking history, primary tumor site, metastatic organ count, neutrophil-to-lymphocyte ratio (NLR), adverse event, sites of distant metastasis, microsatellite instability status, TMB level, line of immunotherapy, and immunotherapy regimen were meticulously recorded. Progression-free survival (PFS) was defined as the duration from the initiation of immunotherapy to the first occurrence of mCRC relapse, disease advancement, or mortality. Radiographic responses were evaluated using computed tomography or magnetic resonance imaging scans at baseline, 6–8 weeks after the initiation of treatment, and bimonthly thereafter, in accordance with the modified RECIST 1.1 for immune based therapeutics (termed iRECIST). The efficacy of immunotherapy was evaluated by categorizing tumor response as complete response (CR), partial response (PR), stable disease (SD), or progressive disease (PD). If immune unconfirmed progressive disease was identified, patients were re-evaluated after 4–6 weeks to confirm whether it was immune confirmed progressive disease (iCPD). iCPD was defined as PD according to the iRECIST criteria.

### 2.3 Statistical analysis

Categorical variables were analyzed using Pearson’s chi-square test, while optimal cut-offs for continuous variables (age: 45 years; BMI: 23.32 kg/m²; NLR: 3.2) were determined based on PFS data using X-tile software(**Supplementary Figure 1**) (8). Univariable Cox regression was employed to assess relationships between baseline characteristics and PFS, with significantly associated variables (*P* < 0.05) subsequently included in a multivariable Cox model to identify independent prognostic factors. To evaluate consistency across patient subgroups and address potential confounding, we performed stratified analyses using both univariable and multivariable Cox regression. All statistical analyses were conducted using IBM SPSS Statistics version 26.0 (IBM, Armonk, NY, USA), with a PFS predictive nomogram developed in R version 3.5.1 incorporating metastatic organ count, immunotherapy line, and tumor mutational burden. A two-sided *P* < 0.05 was considered statistically significant.

## 3 Results

### 3.1 Patient characteristics

**Figure 1:**
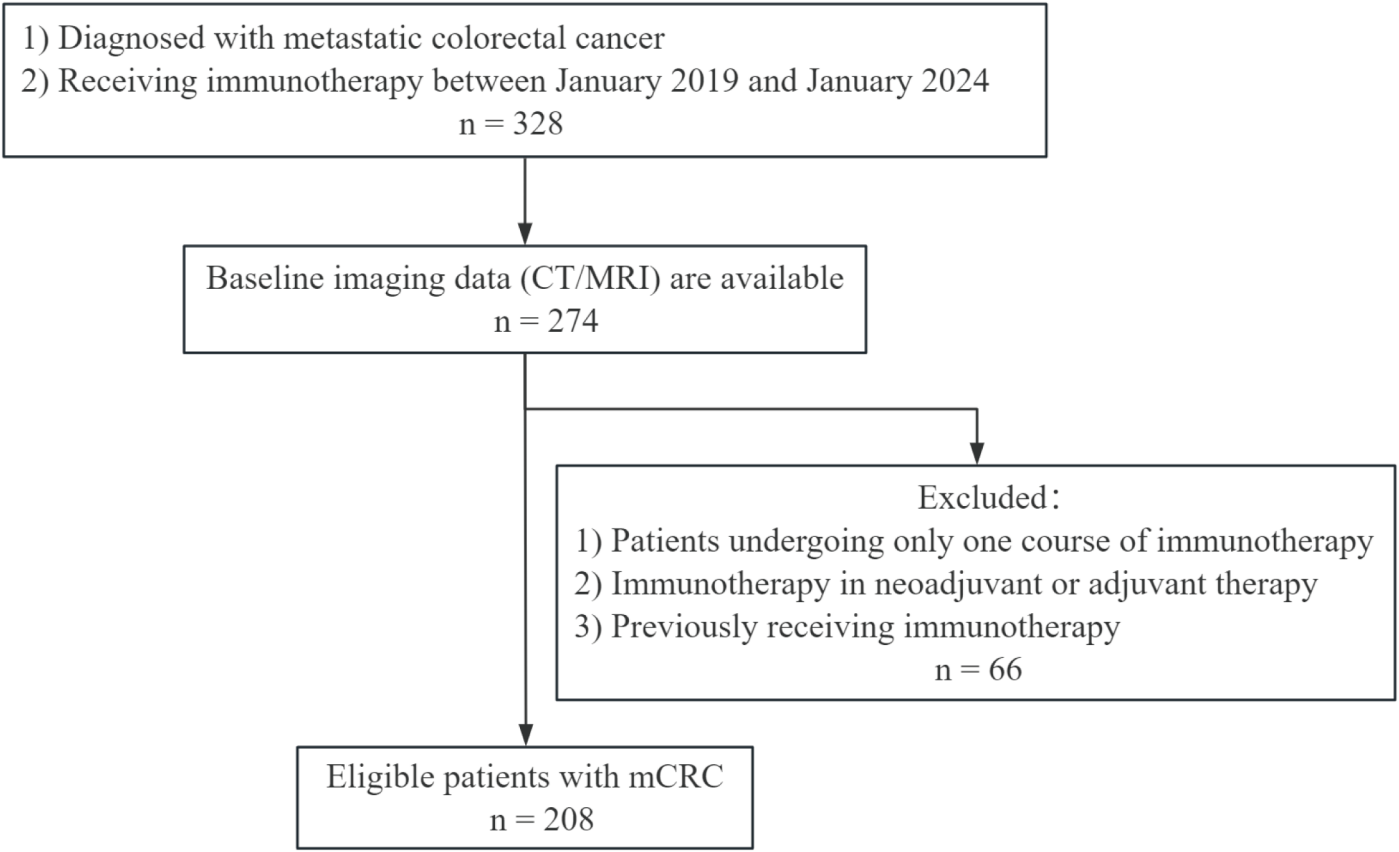
Workflow of patients with mCRC receiving immunotherapy. mCRC, metastatic colorectal cancer.

Our study included 328 patients with mCRC who were receiving immunotherapy administered either as a solo therapy or in combination with other treatment modalities. The immunotherapy agents included a range of PD-1 inhibitors such as pembrolizumab, tirelizumab, nivolumab, camrelizumab, and teripulimab. After applying our eligibility criteria, 208 patients were selected for analysis (**Figure 1**). Patient age ranged from 24 to 85 years (median, 51 years). The cohort consisted of 132 men (63.5%) and 76 women (36.5%). The median follow-up time was 22.2 months. There were no significant differences in demographic and clinical characteristics between patients with and without objective response rates (ORRs), except for ECOG PS, primary tumor site, number of metastatic organs, microsatellite status, TMB level, line of immunotherapy, and immunotherapy regimen (**Table 1**).

At baseline, the metastatic sites among the 208 patients were distributed as follows: liver in 132 (63.5%), lung in 89 (42.8%), distant lymph nodes in 88 (42.3%), peritoneum in 61 (29.3%), ovary in 12 (5.8%), bone in 9 (4.3%), adrenal glands in 8 (3.8%), pleura in 5 (2.4%), brain in 3 (1.4%), other GI sites in 3 (1.4%), spleen in 3 (1.4%), uterus in 2 (0.96%) and pancreas in 1 (0.48%). The ORR varied significantly based on metastatic patterns. Patients with bone metastasis exhibited significantly lower ORR than those without (0% vs 34.2%, *P* = 0.032). Conversely, patients with solitary liver metastasis achieved superior ORR compared to those with non-solitary metastatic involvement (57.1% vs 27.7%, *P* = 0.001). Similarly, both lung metastasis (14.6% vs 46.3%, *P* < 0.001) and concurrent liver-lung metastases (10.0% vs 41.9%, *P* < 0.001) were associated with significantly impaired treatment response compared to cases without these metastatic patterns. A similar pattern was observed for the disease control rate (DCR). Patients with liver metastasis (58.3% vs. 77.6%, *P* = 0.005), lung metastasis (51.7% vs. 75.6%, *P* < 0.001), concurrent liver–lung metastases (41.7% vs. 75.0%, *P* < 0.001), and splenic metastasis (0% vs. 66.3%, *P* = 0.04) exhibited significantly poorer DCR (**Supplementary Table 1**).

Overall, these findings indicate that bone metastasis, non-isolated liver metastasis, lung metastasis, and concurrent liver–lung metastases are associated with unfavorable ORR, whereas liver metastasis, lung metastasis, splenic metastasis, and concurrent liver–lung metastases are associated with poorer DCR in patients with mCRC undergoing immunotherapy.

### 3.2 Independent risk factors for PFS and overall survival in patients with mCRC

To evaluate the correlation between clinicopathological factors and PFS in patients with mCRC receiving immunotherapy, univariable Cox regression analysis was conducted. Age, ECOG PS, metastatic organ count, NLR, microsatellite status, TMB level, and line of immunotherapy were identified as possible predictors of PFS. Subsequent multivariable Cox regression analysis showed that low TMB level (hazard ratio [HR]=2.49, 95% confidence interval [CI]: 1.60–3.88, *P<*0.001), second-or-higher-line immunotherapy (HR = 2.83, 95% CI: 1.81–4.44, *P<*0.001), and high metastatic organ count (3 organs: HR = 2.21, 95%CI: 1.40–3.51, *P=*0.001; ≥ 4 organs: HR = 3.85, 95% CI: 2.06–7.22, *P<*0.001) were independently associated with reduced PFS time in patients with mCRC receiving immunotherapy (**Table 2**). Regarding overall survival (OS), high metastatic organ count, second-or-higher-line immunotherapy, and combination immunotherapy were identified as independent prognostic variables (**Supplementary Table 2**).

**Table 2:** Univariable and multivariable Cox regression analyses identifying risk factors for progression-free survival.

Variables	Univariable HR (95%CI)	*P* value	Multivariable HR (95% CI)	*P* value
Age, years				
≤ 45	1.00			
> 45	1.56 (1.07–2.30)	0.022		
Sex				
Male	1.00			
Female	1.12 (0.80–1.56)	0.515		
BMI				
≤ 23.32	1.00			
> 23.32	1.36 (0.99–1.88)	0.059		
Lynch syndrome				
Yes	0.33 (0.10–1.02)	0.055		
No	1.00			
ECOG PS				
0	1.00			
≥ 1	1.64 (1.18–2.27)	0.003		
Smoker				
Yes	1.02 (0.69–1.49)	0.940		
No	1.00			
Location of primary tumor				
Left	1.00			
Right	0.79 (0.55–1.13)	0.192		
Both	0.74 (0.27–2.02)	0.558		
Metastatic organ count				
1	1.00		1.00	
2	1.95 (1.28–2.97)	0.002	1.40 (0.91–2.16)	0.130
3	3.31 (2.12–5.18)	0.000	2.21 (1.40–3.51)	0.001
≥ 4	7.94 (4.32–14.58)	0.000	3.85 (2.06–7.22)	0.000
Neutrophil-lymphocyte ratio (NLR)				
≤ 3.2	1.00			
> 3.2	0.70 (0.50–0.98)	0.037		
Adverse events				
Yes	1.01 (0.72–1.42)	0.951		
No	1.00			
Microsatellite status				
MSS/MSI-L	1.00			
MSI-H	0.23 (0.13–0.40)	0.000		
Tumor mutation burden (Mb)				
< 10	3.40 (2.22–5.21)	0.000	2.49 (1.60–3.88)	0.000
≥ 10	1.00		1.00	
Line of immunotherapy				
First-line	1.00		1.00	
Second-or-higher-line	4.04 (2.65–6.16)	0.000	2.83 (1.81–4.44)	0.000
Immunotherapy regimen				
Solo therapy	1.00			
Combination therapy	2.71 (1.56–4.72)	0.000		

Variables are expressed as n (%).

BMI, body mass index; CI, confidence interval; ECOG PS, Eastern Cooperative Oncology Group Performance Status; HR, hazard ratio; mCRC, metastatic colorectal cancer; MSI-H, microsatellite instability-high; MSI-L, microsatellite instability-low; MSS, microsatellite stable.

To address potential confounding effects arising from MSI status and TMB levels, we initially summarized the clinicopathological characteristics associated with MSI-H or high TMB level (**Supplementary Table 3**). Our analysis revealed that patients exhibiting MSI-H or elevated TMB levels were predominantly characterized by younger age (<45 years), fewer metastatic organ involvement, higher likelihood of receiving first-line immunotherapy, and increased proportion of immunotherapy-only treatment regimens. Subsequent univariable and multivariable Cox regression analyses were performed in **Supplementary Tables 4, 5** to evaluate PFS and OS within the MSI-H/high TMB subgroup. These analyses identified metastatic organ count as the sole independent prognostic factor for both PFS and OS in this subgroup. Further stratification analyses in **Supplementary Tables 6, 7** focused on the MSI-L/MSS with low TMB subgroup. The Cox regression models demonstrated that metastatic organ count and line of immunotherapy emerged as independent prognostic factors for PFS, while only line of immunotherapy maintained independent prognostic significance for OS.

**Figure 2:**
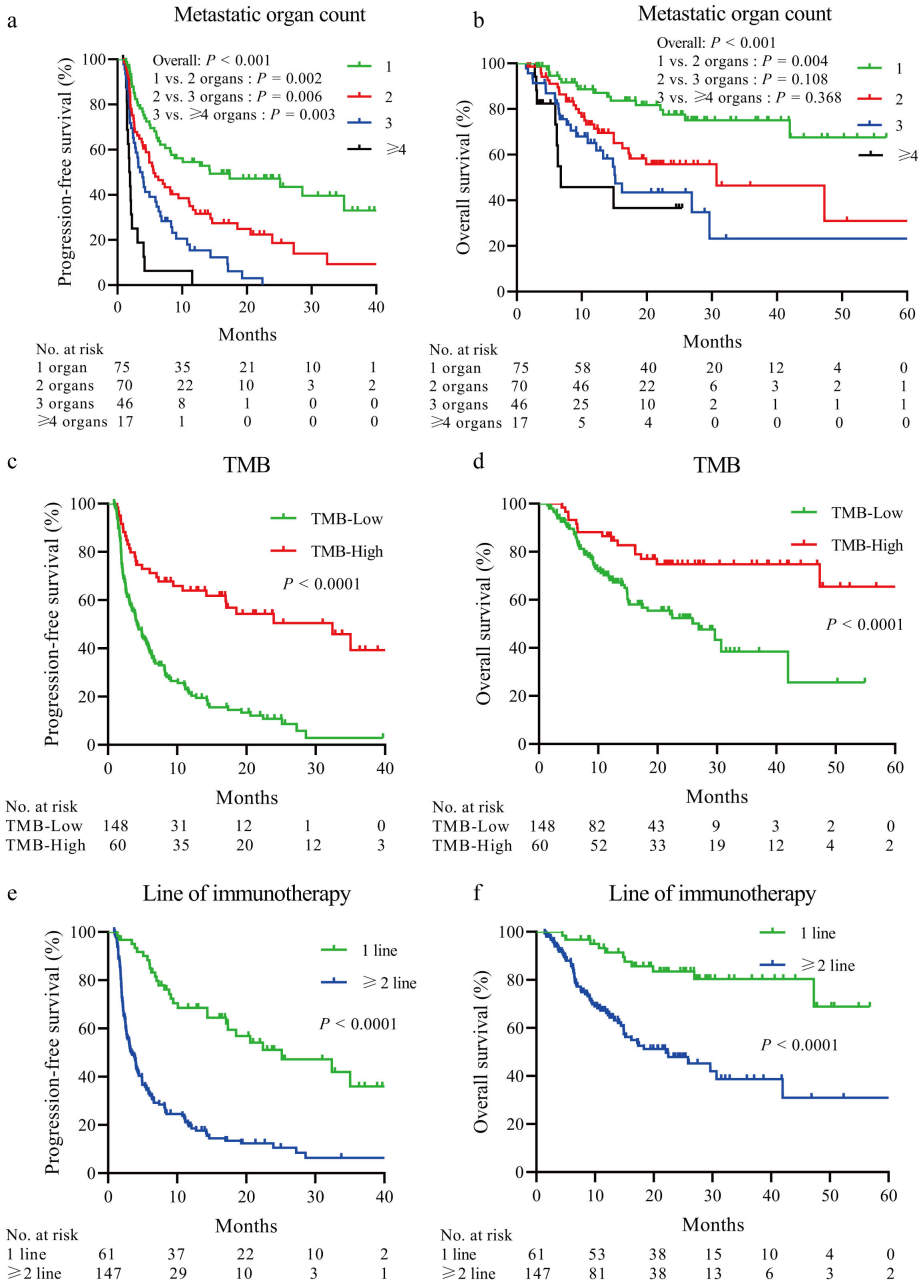
Kaplan–Meier plots depicting the association of PFS and OS with metastatic organ count, TMB, and line of immunotherapy in patients with mCRC. **(A, B)** The higher the count of metastatic organs, the poorer the PFS and OS, as demonstrated by the log-rank test. **(C–F)**. Patients with low TMB levels or those receiving second-line or later immunotherapy exhibited significantly poorer PFS and OS, with statistical significance indicated by the log-rank test. mCRC, metastatic colorectal cancer; OS, overall survival; PFS, progression-free survival; TMB, tumor mutational burden.

**Figure 3:**
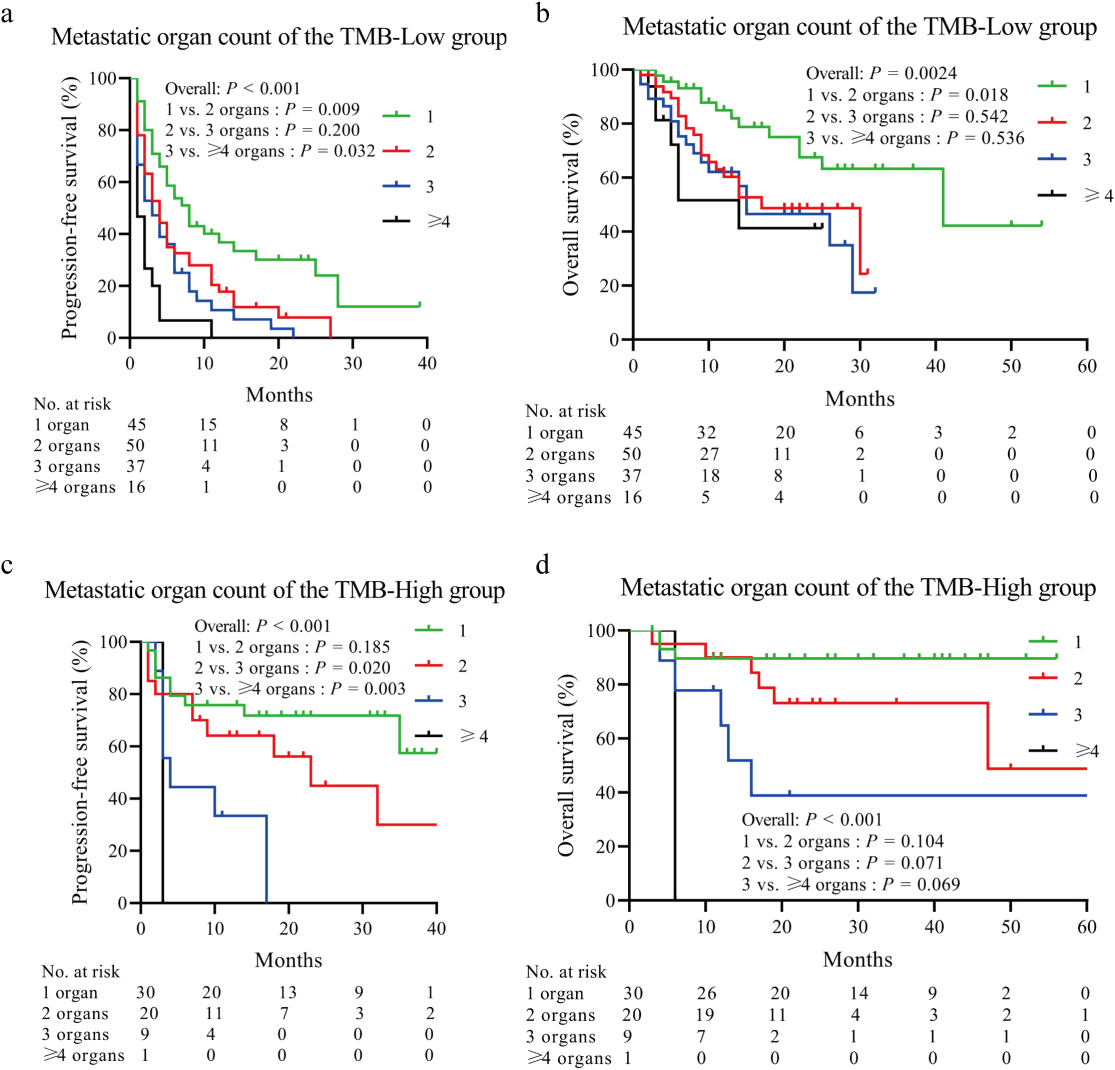
Kaplan–Meier plots of PFS and OS stratified by TMB levels. **(A, B)** In the TMB-low group, an increased metastatic organ count was significantly associated with reduced PFS and OS. **(C, D)** In the TMB-high group, a higher metastatic organ count also reliably identified patients with a less favorable prognosis. OS, overall survival; PFS, progression-free survival; TMB, tumor mutational burden.

**Figure 4:**
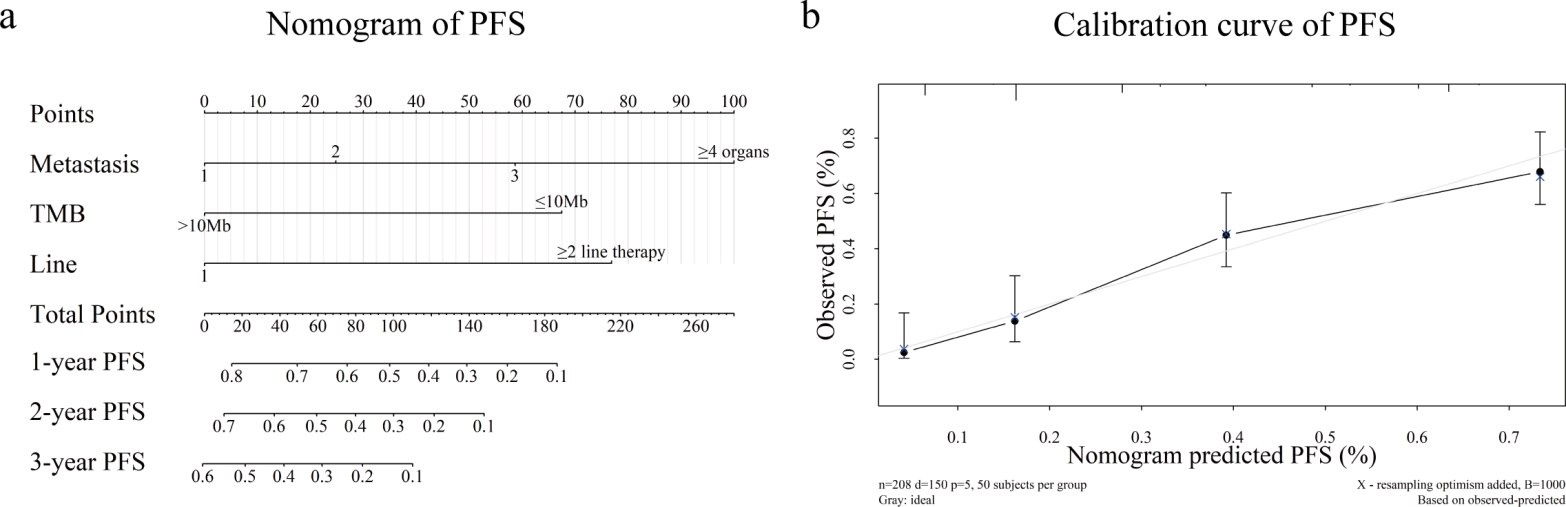
**(A)** A nomogram developed for estimating the probability of PFS in patients with mCRC, taking into account the number of metastatic organs, TMB levels, and the line of therapy. **(B)** The calibration curve depicting the accuracy of the nomogram in predicting the 1-year PFS for patients. mCRC, metastatic colorectal cancer; PFS, progression-free survival; TMB, tumor mutational burden.

Kaplan–Meier curve analysis showed that patients with increased metastatic organ counts experienced significantly worse PFS and OS (**Figures 2A, B**). Patients with low TMB levels or those receiving second-or-higher-line immunotherapy also had significantly reduced PFS and OS times (all *P* < 0.0001) (**Figures 2C–F**). The negative effect of high metastatic organ count on PFS and OS was observed in those with high and low TMB levels (**Figure 3**). To further quantify the combined prognostic impact of these variables, we developed a nomogram integrating metastatic organ count, line of immunotherapy, and TMB level to predict individualized probabilities of PFS (**Figure 4A**). Each variable was assigned a specific point value according to its relative contribution to prognosis, and the total score corresponded to the estimated 1-, 2-, and 3-year PFS probabilities. The calibration plot (**Figure 4B**) demonstrated excellent agreement between predicted and observed PFS outcomes, indicating that the model is well-calibrated and highly accurate.

### 3.3 Relationship between tumor response to immunotherapy and metastatic organ count

The correlation between metastatic organ count and immunotherapy response is detailed in **Table 3**. Analyses based on the iRECIST criteria reveal that the metastatic organ count is closely associated with the efficacy of immunotherapy. As the number of metastatic organs gradually increases from one to four or more, the ORR sharply drops from 49.3% to 5.9%, and the DCR also significantly decreases from 80.0% to 29.4%, with statistically significant differences (*P* < 0.0001). This clearly demonstrates that an increase in the metastatic organ count significantly reduces the effectiveness of immunotherapy.

**Table 3:** Relationship between tumor response and metastatic organ count in patients with mCRC receiving immunotherapy.

Treatment response	Metastatic organ count				*P* value
	1 (n = 75)	2 (n = 70)	3 (n = 46)	≥ 4 (n = 17)	
CR	11 (14.7)	3 (4.3)	0 (0.0)	0 (0.0)	
PR	27 (36.0)	18 (25.7)	9 (19.6)	1 (5.9)	
SD	23 (30.7)	24 (34.3)	17 (37.0)	4 (23.5)	
PD	14 (18.7)	25 (35.7)	20 (43.5)	12 (70.6)	
ORR (CR + PR)	37 (49.3)	21 (30.0)	9 (19.6)	1 (5.9)	0.000
DCR (CR + PR + SD)	60 (80.0)	45 (64.3)	26 (56.5)	5 (29.4)	0.000

Variables are expressed as number of patients (%).

CR, complete response; DCR, disease control rate; mCRC, metastatic colorectal cancer; ORR, objective response rate; PD, progressive disease; PR, partial response; SD, stable disease.

### 3.4 Subgroup analyses by monotherapy and combination therapy

To assess the consistency of the prognostic value of metastatic organ count across different therapeutic contexts, we conducted subgroup analyses stratified by treatment type (monotherapy vs. combination therapy; **Supplementary Tables 8, 9**).

In the monotherapy cohort, the type of PD-1 inhibitor (including Nivolumab, Pembrolizumab, Sintilimab, Toripalimab, Tislelizumab and Camrelizumab) showed no significant association with PFS in univariable or multivariable analyses (all *P* > 0.05). By contrast, the presence of three metastatic organs was independently associated with significantly shorter PFS compared to fewer sites (HR = 11.62, 95% CI: 2.22–60.98, *P* = 0.004), confirming metastatic organ count as a robust prognostic factor in patients with mCRC receiving monotherapy.

Within the combination therapy subgroup, regimens were categorized as immunotherapy with chemotherapy, with targeted therapy, or with both, due to substantial heterogeneity. Here, neither the specific treatment category nor the PD-1 inhibitor type significantly influenced PFS. Instead, multivariable analyses identified the line of immunotherapy, TMB, and metastatic organ count as independent predictors. Second-line or later immunotherapy was associated with worse PFS compared to first-line treatment (HR = 3.04, 95% CI: 1.84–5.00, P < 0.001). Similarly, low TMB (<10 mutations/Mb) predicted shorter PFS (HR = 2.44, 95% CI: 1.45–4.13, *P* = 0.001). Most notably, increasing metastatic organ count remained strongly associated with poorer PFS, with HR of 1.98 (95% CI: 1.21–3.26, *P* = 0.007) for three metastatic organ and 3.55 (95% CI: 1.85–6.81, *P* < 0.001) for four or more metastatic organ, using a single metastatic organ as the reference.

Collectively, these subgroup analyses reinforce that the negative prognostic impact of a higher metastatic organ count on PFS is consistent, irrespective of the type of PD-1 inhibitor or treatment regimen used.

## 4 Discussion

Administration of immunotherapy is a significant milestone in oncology, revolutionizing the therapeutic landscape for patients with mCRC. The proliferation of clinical trials exploring immunotherapy across various treatment settings, ranging from adjuvants to multiple lines of therapy, reflects its growing importance in cancer care (9). However, the molecular heterogeneity inherent in mCRC results in considerable variability in treatment responses, even in patients with a similar TMB level or MSI status. This variability highlights the pressing need for robust prognostic markers to improve immunotherapeutic strategies.

Our study is built upon the existing literature by examining the role of metastatic organ count as a predictive factor for immunotherapy response in mCRC. MSI status (10, 11) and TMB level (12) are well-established biomarkers of immunotherapy response in CRC. These findings are corroborated by our study findings. However, responses have been observed in patients lacking these characteristics (13); conversely, some patients with high MSI or TMB levels do not respond to immunotherapy (10). Furthermore, the assessment of these biomarkers often requires complex, costly, and time-consuming analyses.

Predictive markers for immunotherapy have been extensively studied in solid tumors, with research suggesting a possible correlation between metastatic organ count and treatment response in melanoma immunotherapy (14). Our findings align with this, showing a significant inverse relationship between metastatic organ count and ORR or DCR in patients with mCRC receiving immunotherapy. Multivariable Cox analysis further confirmed that increased metastatic organ count was an independent factor associated with short PFS time in these patients. Therefore, metastatic organ count should be integrated with MSI/TMB status in clinical decision-making, as it reflects tumor biological aggression through distinct mechanisms; MSI/TMB status captures immunogenic potential, while metastatic is different from that.

The efficacy of immunotherapy may be primarily influenced by metastatic organ count through the following two aspects: tumor burden and the diversity of microenvironments.

First, metastatic organ count is typically associated with tumor burden, a critical factor in assessing prognosis and immune response across various cancers (15). For instance, in advanced non-small-cell lung cancer (NSCLC), a substantial tumor burden is recognized as an independent prognostic factor that affects the outcome of immunotherapy (16). Second, each metastatic organ presents a unique intrinsic immune microenvironment, potentially including immunosuppressive stroma that differs from that of primary tumor. Increased metastatic organ count suggests a more complex and diverse immune contexture, which may induce resistance to immunotherapy. Primary tumor facilitates metastasis by inducing a supportive microenvironment at secondary sites, known as premetastatic niches (17, 18). In a novel mouse model of spontaneous multiorgan metastases in CRC with MSI-H, peritoneal metastases were found to lack tertiary lymphoid structures, which are crucial for housing B and T cells with functional activity. The absence of these structures can contribute to diminished immunotherapy efficacy (19). Furthermore, the composition of microbial communities within metastatic tumors varies across different organs and is linked to cancer-related phenomena such as hypoxia and inflammation. An increased number of metastatic organs correlates with the more complex of microbiome involved. These microbial communities can modulate immune cell infiltration and immunotherapy efficacy. For example, *Fusobacterium*, which is negatively associated with responsiveness to immunotherapy in NSCLC, has also been implicated in the progression of colorectal cancer (20). It can interact directly with tumor-infiltrating lymphocytes, suppressing their activity. Additionally, the dynamic microbial-host interface in the human respiratory tract indicates that lung microbial communities can significantly influence local immune responses, further emphasizing the multifactorial nature of immunotherapy resistance (21).

This nuanced understanding of the interplay between metastatic organ count, tumor microenvironment, and microbiome underscores the need for a comprehensive approach to evaluating and optimizing immunotherapy strategies for patients with mCRC.

In our current research on mCRC, we closely examined the impact of immunotherapy across different metastatic sites. Our primary findings indicated that the response to immunotherapy varied significantly depending on the organs affected by metastasis. Specifically, patients with liver-only metastasis, distant lymph node metastasis, and ovarian metastasis demonstrated a more positive response to immunotherapy. Conversely, those with lung metastasis, especially when accompanied by liver involvement, had a relatively poor outcome. The suboptimal response to immunotherapy observed in patients with lung metastases may be attributed not only to the influence of the lung microbiome but also to the infiltration of immunosuppressive cells within pulmonary tissues. In pulmonary metastatic nodules, natural killer (NK) cells are predominantly composed of the CD27^high^ subset, which exhibits a relatively undifferentiated phenotype. These cells show low expression of CD11b, KLRG1, and granzyme B (Gzmb), and undergo a phenotypic shift toward a TGF-β–driven state, resulting in impaired persistence and function (22). TGF-β further amplifies local immunosuppression by promoting the differentiation and expansion of regulatory T cells and enhancing their suppressive activity. Concurrently, TGF-β upregulates vascular endothelial growth factor A expression, establishing a positive feedback loop that fosters tumor angiogenesis and reinforces an immunosuppressive microenvironment (23).

However, a secondary analysis of regorafenib, nivolumab (REGONIVO) or regorafenib, ipilimumab, and nivolumab (RIN) (24) revealed a distinct pattern in patients with MSS mCRC. Contrary to our initial observations, lung metastasis in the MSS mCRC group showed the highest ORR to immunotherapy, while liver and peritoneal metastases had lower ORRs. Moreover, the incidence of lung metastasis in this secondary analysis was over two times that of liver metastasis, a result that deviated from previous research. We suspect that this difference might be due to the selection criteria in the REGONIVO-related data, potentially introducing a selection bias. It’s important to note that our original study included all mCRC patients undergoing immunotherapy, regardless of their MSI-H or MSS status. This comprehensive approach ensured that our results were more reflective of the real-world clinical situations, offering a broader perspective on the efficacy of immunotherapy for mCRC patients.

This study has some limitations. First, its retrospective and single-center design, together with a relatively limited sample size, may restrict the generalizability of the findings. These factors highlight the need for larger, prospective, multicenter studies to further validate our results. Second, our focus was primarily on the response rate of overall tumor burden to immunotherapy, without a detailed analysis of the treatment responses across different metastatic sites post-immunotherapy. Third, owing to the limited sample size, a comprehensive Cox proportional hazards analysis stratified by metastatic organ was not performed. Finally, as this was a retrospective study, most patients did not undergo cytokine/chemokine profiling or immune cell population analysis. The absence of these immunological data introduces potential bias and limits our ability to explore the underlying immune mechanisms associated with therapeutic outcomes.

## 5 Conclusions

In summary, our study highlights the critical influence of metastatic organ count on immunotherapy outcomes in patients with mCRC. An increased metastatic organ count is associated with reduced immunotherapy ORR and worse prognosis, with particularly adverse outcomes in patients with lung metastasis (especially concurrent lung and liver metastases). These findings contribute to a growing body of evidence that supports the use of metastatic organ count as a pragmatic prognostic marker in the immunotherapy treatment paradigm for mCRC.

## Data Availability

Publicly available datasets were analyzed in this study. This data can be found here: The raw data used in this study will be uploaded to the Research Data Deposit (www.researchdata.org.cn).
