# Supplementary material for: Metastatic organ count stratifies survival in immunotherapy-treated metastatic colorectal cancer: a retrospective cohort study
Source: Front Immunol. 2025 Dec 9;16:1679041. doi: 10.3389/fimmu.2025.1679041 (PMC12723019; doi:10.3389/fimmu.2025.1679041)
Supplement: Supplementary file 1 [file DataSheet1.docx]

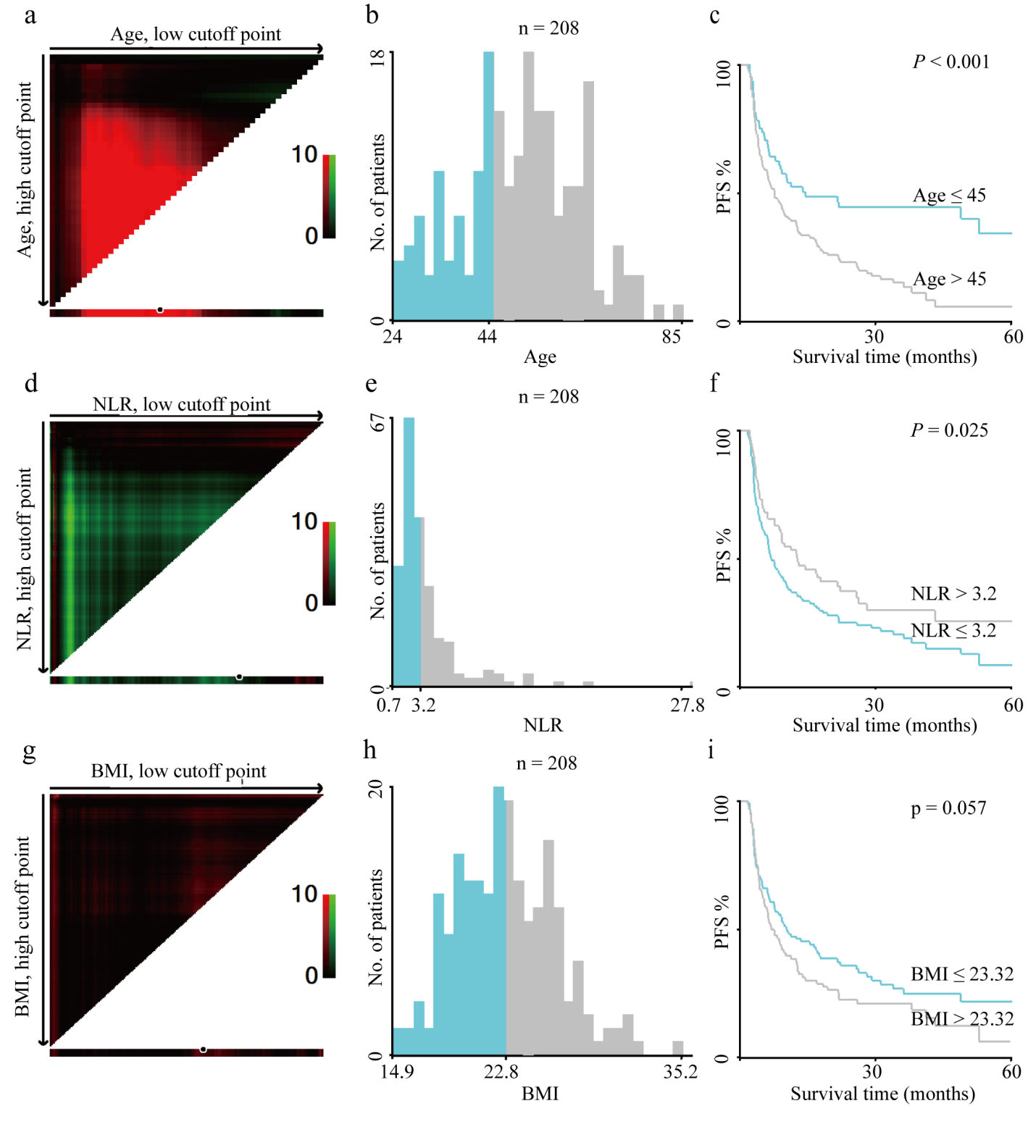


**Supplemental Fig. S1** X-tile analysis for determining optimal prognostic cutoffs of age, NLR, and BMI in mCRC patients receiving immunotherapy. Heatmaps display chi-square (χ²) values from log-rank tests across all possible cutoffs. Red indicates values associated with poorer PFS (higher risk), green with better PFS (lower risk). The brightest colored regions (peak χ² values) correspond to optimal cutoffs, marked with white circles. BMI: body mass index; NLR: neutrophil-to-lymphocyte ratio; CRC: colorectal cancer.

**Supplemental Table 1**. Relationship between tumor response and metastatic organ distribution in patients with mCRC receiving immunotherapy

| Metastatic organs | n | ORR, n (%) | *P* value | DCR, n (%) | *P* value | PD, n (%) |
| --- | --- | --- | --- | --- | --- | --- |
| Adrenal gland | 8 | 0 (0.0) | 0.055^a^ | 4 (50.0) | 0.452^a^ | 4 (50.0) |
| Bone | 9 | 0 (0.0) | 0.032^a^ | 3 (33.3) | 0.067^a^ | 6 (66.7) |
| Brain | 3 | 0 (0.0) | 0.552^a^ | 2 (66.7) | 1.00^a^ | 1 (33.3) |
| Liver | 132 | 40 (30.3) | 0.333 | 77 (58.3) | 0.005 | 55 (42.7) |
| Liver-only metastasis | 35 | 20 (57.1) | 0.001 | 27 (77.1) | 0.109 | 8 (22.9) |
| Liver and other non-lung metastases | 37 | 14 (37.8) | 0.462 | 25 (67.6) | 0.758 | 12 (32.4) |
| Lung | 89 | 13 (14.6) | 0.000 | 46 (51.7) | 0.000 | 43 (48.3) |
| Lung-only metastasis | 9 | 2 (22.2) | 0.721 | 6 (66.7) | 1.00^a^ | 3 (33.3) |
| Lung and other non-liver metastases | 20 | 5 (25.0) | 0.440 | 15 (75.0) | 0.342 | 5 (25.0) |
| Lung and liver metastases | 60 | 6 (10.0) | 0.000 | 25 (41.7) | 0.000 | 35 (58.3) |
| Lymph node^b^ | 88 | 27 (30.7) | 0.597 | 58 (65.9) | 0.892 | 30 (34.1) |
| Other GI sites | 3 | 1 (33.3) | 1.000^a^ | 1 (33.3) | 0.275^a^ | 2 (66.7) |
| Ovary | 12 | 5 (41.7) | 0.533 | 5 (41.7) | 0.533 | 7 (58.3) |
| Pancreas | 1 | 0 (0.0) | 1.000^a^ | 0 (0.0) | 0.346^a^ | 1 (100.0) |
| Peritoneum | 61 | 18 (29.5) | 0.528 | 38 (62.3) | 0.546 | 23 (37.7) |
| Pleura | 5 | 0 (0.0) | 0.175^a^ | 3 (60.0) | 1.00^a^ | 2 (40.0) |
| Spleen | 3 | 0 (0.0) | 0.552^a^ | 0 (0.0) | 0.040^a^ | 3 (100.0) |
| Uterus | 2 | 2 (100.0) | 0.106^a^ | 2 (100.0) | 0.545^a^ | 0 (0.0) |
| Others | 15 | 4 (26.7) | 0.778^a^ | 11 (73.3) | 0.502 | 4 (26.7) |
| Total | 208 | 68 (32.7) |  | 136 (65.4) |  | 72 (34.6) |

^a^ Fisher’s exact test. Variables are expressed as n (%).

^b^ The lymph node metastasis referred to here is distant lymph node metastasis, not regional lymph node metastasis.

CR, complete response; GI, gastrointestinal duct; mCRC, metastatic colorectal cancer; ORR, objective response rate; PR, partial response.

**Supplemental Table 2**. Univariable and multivariable Cox regression analyses to identify the risk factors for overall survival

| Variables | Univariable  HR (95% CI) | *P* value | Multivariable  HR (95% CI) | *P* value |
| --- | --- | --- | --- | --- |
| Age, years |  |  |  |  |
| ≤ 45 | 1.00 |  |  |  |
| > 45 | 1.83 (1.04–3.22) | 0.035 |  |  |
| Sex |  |  |  |  |
| Male | 1.00 |  |  |  |
| Female | 0.86 (0.69–1.09) | 0.214 |  |  |
| BMI |  |  |  |  |
| ≤ 23.32 | 1.00 |  |  |  |
| > 23.32 | 1.03(0.64–1.65) | 0.908 |  |  |
| Lynch syndrome |  |  |  |  |
| Yes | 0.43 (0.11–1.77) | 0.243 |  |  |
| No | 1.00 |  |  |  |
| ECOG PS |  |  |  |  |
| 0 | 1.00 |  |  |  |
| ≥ 1 | 2.08 (1.31–3.28) | 0.002 |  |  |
| Smoker |  |  |  |  |
| Yes | 1.03 (0.60–1.78) | 0.905 |  |  |
| No | 1.00 |  |  |  |
| Location of primary tumor |  |  |  |  |
| Left | 1.00 |  |  |  |
| Right | 0.75 (0.45–1.24) | 0.260 |  |  |
| Both | 0.35 (0.05–2.54) | 0.300 |  |  |
| Metastatic organ count |  |  |  |  |
| 1 | 1.00 |  | 1.00 |  |
| 2 | 2.42 (1.30–4.52) | 0.005 | 1.73 (0.91–3.31) | 0.096 |
| 3 | 3.76 (1.97–7.18) | 0.000 | 2.48 (1.26–4.85) | 0.008 |
| ≥ 4 | 5.28 (2.23–12.51) | 0.000 | 2.78 (1.13–6.82) | 0.026 |
| Neutrophil-lymphocyte ratio (NLR) |  |  |  |  |
| ≤ 3.2 | 1.00 |  |  |  |
| > 3.2 | 1.03 (0.65–1.65) | 0.893 |  |  |
| Adverse events |  |  |  |  |
| Yes | 0.87 (0.54–1.39) | 0.557 |  |  |
| No | 1.00 |  |  |  |
| Microsatellite status |  |  |  |  |
| MSS/MSI-L | 1.00 |  |  |  |
| MSI-H | 0.30 (0.14–0.64) | 0.002 |  |  |
| Tumor mutation burden (Mb) |  |  |  |  |
| < 10 | 2.60 (1.45–4.64) | 0.001 |  |  |
| ≥ 10 | 1.00 |  |  |  |
| Line of immunotherapy |  |  |  |  |
| First-line | 1.00 |  | 1.00 |  |
| Second-or-higher-line | 4.04 (2.65–6.16) | 0.000 | 2.79 (1.40–5.54) | 0.004 |
| Immunotherapy regimen |  |  |  |  |
| Solo therapy | 1.00 |  | 1.00 |  |
| Combination therapy | 3.76 (1.51–9.35) | 0.004 | 2.71 (1.07–6.89) | 0.036 |

Variables are expressed as n (%).

BMI, body mass index; CI, confidence interval; ECOG PS, Eastern Cooperative Oncology Group Performance Status; HR, hazard ratio; MSI-H, microsatellite instability-high; MSI-L, microsatellite instability-low; MSS, microsatellite stable.

**Supplemental Table 3**. Relationship between MSI-H or high TMB level status and important clinical indicators

| Variables | MSI-H or high TMB level, n (%) | | *P* value |
| --- | --- | --- | --- |
|  | Yes | No |  |
| Age, years |  |  | 0.018 |
| ≤ 45 | 24 (39.3) | 34 (23.1) |  |
| > 45 | 37 (60.7) | 113 (76.9) |  |
| ECOG PS |  |  | 0.105 |
| 0 | 43 (70.5) | 86 (58.5) |  |
| ≥ 1 | 18 (29.5) | 61 (41.5) |  |
| Metastatic organ count |  |  | 0.008 |
| 1 | 31 (50.8) | 44 (29.9) |  |
| 2 | 20 (32.8) | 50 (34.0) |  |
| 3 | 9 (14.8) | 37 (25.2) |  |
| ≥ 4 | 1 (1.6) | 16 (10.9) |  |
| Line of immunotherapy |  |  | 0.000 |
| First-line | 33 (54.1) | 28 (19.0) |  |
| Second-or-higher-line | 28 (45.9) | 119 (81.0) |  |
| Immunotherapy regimen |  |  | 0.000 |
| Solo therapy | 24 (39.3) | 6 (4.1) |  |
| Combination therapy | 37 (60.7) | 141 (95.9) |  |

ECOG PS, Eastern Cooperative Oncology Group Performance Status; MSI-H, microsatellite instability-high; TMB, Tumor mutation burden.

**Supplemental Table 4**. Univariable and multivariable Cox regression analyses to identify the risk factors for progression-free survival of the MSI-H or high TMB subgroup

| Variables | Univariable  HR (95% CI) | *P* value | Multivariable  HR (95% CI) | *P* value |
| --- | --- | --- | --- | --- |
| Age, years |  |  |  |  |
| ≤ 45 | 1.00 |  |  |  |
| > 45 | 1.58 (0.70–3.53) | 0.269 |  |  |
| Sex |  |  |  |  |
| Male | 1.00 |  |  |  |
| Female | 1.07 (0.50–2.30) | 0.857 |  |  |
| BMI |  |  |  |  |
| ≤ 23.32 | 1.00 |  |  |  |
| > 23.32 | 1.13 (0.48–2.66) | 0.783 |  |  |
| Lynch syndrome |  |  |  |  |
| Yes | 0.78 (0.23–2.58) | 0.678 |  |  |
| No | 1.00 |  |  |  |
| ECOG PS |  |  |  |  |
| 0 | 1.00 |  |  |  |
| ≥ 1 | 2.59 (1.19–5.64) | 0.017 |  |  |
| Smoker |  |  |  |  |
| Yes | 1.05 (0.46–2.38) | 0.909 |  |  |
| No | 1.00 |  |  |  |
| Location of primary tumor |  |  |  |  |
| Left | 1.00 |  |  |  |
| Right | 0.62 (0.28–1.40) | 0.248 |  |  |
| Both | 0.84 (0.19–3.63) | 0.811 |  |  |
| Metastatic organ count |  |  |  |  |
| 1 | 1.00 |  | 1.00 |  |
| 2 | 1.92 (0.78–4.73) | 0.158 | 1.92 (0.78–4.73) | 0.158 |
| 3 | 5.13 (1.89–13.94) | 0.001 | 5.13 (1.89–13.94) | 0.001 |
| ≥ 4 | 115.02 (6.74–1964.40) | 0.001 | 115.02 (6.74–1964.40) | 0.001 |
| Neutrophil-lymphocyte ratio (NLR) |  |  |  |  |
| ≤ 3.2 | 1.00 |  |  |  |
| > 3.2 | 1.00 (0.48–2.11) | 0.993 |  |  |
| Adverse events |  |  |  |  |
| Yes | 0.74 (0.35–1.57) | 0.432 |  |  |
| No | 1.00 |  |  |  |
| Line of immunotherapy |  |  |  |  |
| First-line | 1.00 |  |  |  |
| Second-or-higher-line | 2.57 (1.19–5.55) | 0.016 |  |  |
| Immunotherapy regimen |  |  |  |  |
| Solo therapy | 1.00 |  |  |  |
| Combination therapy | 1.41 (0.65–3.07) | 0.385 |  |  |

Variables are expressed as n (%).

BMI, body mass index; CI, confidence interval; ECOG PS, Eastern Cooperative Oncology Group Performance Status; HR, hazard ratio; mCRC, metastatic colorectal cancer; MSI-H, microsatellite instability-high; TMB, Tumor mutation burden.

**Supplemental Table 5**. Univariable and multivariable Cox regression analyses to identify the risk factors for overall survival of the MSI-H or high TMB subgroup

| Variables | Univariable  HR (95% CI) | *P* value | Multivariable  HR (95% CI) | *P* value |
| --- | --- | --- | --- | --- |
| Age, years |  |  |  |  |
| ≤ 45 | 1.00 |  |  |  |
| > 45 | 2.46 (0.78–7.78) | 0.125 |  |  |
| Sex |  |  |  |  |
| Male | 1.00 |  |  |  |
| Female | 0.74 (0.45–1.21) | 0.236 |  |  |
| BMI |  |  |  |  |
| ≤ 23.32 | 1.00 |  |  |  |
| > 23.32 | 0.22 (0.03–1.63) | 0.137 |  |  |
| Lynch syndrome |  |  |  |  |
| Yes | 0.76 (0.17–3.33) | 0.711 |  |  |
| No | 1.00 |  |  |  |
| ECOG PS |  |  |  |  |
| 0 | 1.00 |  |  |  |
| ≥ 1 | 2.54 (0.92–7.02) | 0.072 |  |  |
| Smoker |  |  |  |  |
| Yes | 0.56 (0.16–1.96) | 0.362 |  |  |
| No | 1.00 |  |  |  |
| Location of primary tumor |  |  |  |  |
| Left | 1.00 |  |  |  |
| Right | 0.51 (0.18–1.48) | 0.217 |  |  |
| Both | 0.00 (0.00) | 0.984 |  |  |
| Metastatic organ count |  |  |  |  |
| 1 | 1.00 |  | 1.00 |  |
| 2 | 2.25 (0.64–7.99) | 0.208 | 2.25 (0.64–7.99) | 0.208 |
| 3 | 5.92 (1.56–22.39) | 0.009 | 5.92 (1.56–22.39) | 0.009 |
| ≥ 4 | 30.61 (2.89–324.19) | 0.004 | 30.61 (2.89–324.19) | 0.004 |
| Neutrophil-lymphocyte ratio (NLR) |  |  |  |  |
| ≤ 3.2 | 1.00 |  |  |  |
| > 3.2 | 1.14 (0.43–3.07) | 0.791 |  |  |
| Adverse events |  |  |  |  |
| Yes | 0.56 (0.21–1.51) | 0.250 |  |  |
| No | 1.00 |  |  |  |
| Line of immunotherapy |  |  |  |  |
| First-line | 1.00 |  |  |  |
| Second-or-higher-line | 1.90 (0.71–5.13) | 0.204 |  |  |
| Immunotherapy regimen |  |  |  |  |
| Solo therapy | 1.00 |  |  |  |
| Combination therapy | 3.47 (0.99–12.19) | 0.053 |  |  |

Variables are expressed as n (%).

BMI, body mass index; CI, confidence interval; ECOG PS, Eastern Cooperative Oncology Group Performance Status; HR, hazard ratio; mCRC, metastatic colorectal cancer; MSI-H, microsatellite instability-high; TMB, Tumor mutation burden.

**Supplemental Table 6**. Univariable and multivariable Cox regression analyses to identify the risk factors for progression-free survival of the MSI-L/MSS with low TMB subgroup

| Variables | Univariable  HR (95% CI) | *P* value | Multivariable  HR (95% CI) | *P* value |
| --- | --- | --- | --- | --- |
| Age, years |  |  |  |  |
| ≤ 45 | 1.00 |  |  |  |
| > 45 | 1.17 (0.76–1.81) | 0.482 |  |  |
| Sex |  |  |  |  |
| Male | 1.00 |  |  |  |
| Female | 1.26 (0.87–1.83) | 0.227 |  |  |
| BMI |  |  |  |  |
| ≤ 23.32 | 1.00 |  |  |  |
| > 23.32 | 1.05 (0.73–1.50) | 0.800 |  |  |
| ECOG PS |  |  |  |  |
| 0 | 1.00 |  |  |  |
| ≥ 1 | 1.25 (0.87–1.79) | 0.228 |  |  |
| Smoker |  |  |  |  |
| Yes | 1.22 (0.78–1.88) | 0.383 |  |  |
| No | 1.00 |  |  |  |
| Location of primary tumor |  |  |  |  |
| Left | 1.00 |  |  |  |
| Right | 1.05 (0.70–1.57) | 0.808 |  |  |
| Both | 4.24 (1.03–17.53) | 0.046 |  |  |
| Metastatic organ count |  |  |  |  |
| 1 | 1.00 |  | 1.00 |  |
| 2 | 1.85 (1.15–2.98) | 0.012 | 1.23 (0.75–2.02) | 0.419 |
| 3 | 2.46 (1.49–4.06) | 0.000 | 1.88 (1.12–3.13) | 0.016 |
| ≥ 4 | 5.25 (2.75–10.01) | 0.000 | 3.26 (1.68–6.34) | 0.000 |
| Neutrophil-lymphocyte ratio (NLR) |  |  |  |  |
| ≤ 3.2 | 1.00 |  |  |  |
| > 3.2 | 0.72 (0.49–1.07) | 0.106 |  |  |
| Adverse events |  |  |  |  |
| Yes | 0.97 (0.66–1.43) | 0.889 |  |  |
| No | 1.00 |  |  |  |
| Line of immunotherapy |  |  |  |  |
| First-line | 1.00 |  | 1.00 |  |
| Second-or-higher-line | 3.80 (2.25–6.44) | 0.000 | 3.24 (1.86–5.64) | 0.000 |
| Immunotherapy regimen |  |  |  |  |
| Solo therapy | 1.00 |  |  |  |
| Combination therapy | 1.60 (0.59–4.33) | 0.360 |  |  |

Variables are expressed as n (%).

BMI, body mass index; CI, confidence interval; ECOG PS, Eastern Cooperative Oncology Group Performance Status; HR, hazard ratio; mCRC, metastatic colorectal cancer; MSI-L, microsatellite instability-low; MSS, microsatellite stable; TMB, Tumor mutation burden.

**Supplemental Table 7**. Univariable and multivariable Cox regression analyses to identify the risk factors for overall survival of the MSI-L/MSS with low TMB subgroup

| Variables | Univariable  HR (95% CI) | *P* value | Multivariable  HR (95% CI) | *P* value |
| --- | --- | --- | --- | --- |
| Age, years |  |  |  |  |
| ≤ 45 | 1.00 |  |  |  |
| > 45 | 1.20 (0.63–2.27) | 0.579 |  |  |
| Sex |  |  |  |  |
| Male | 1.00 |  |  |  |
| Female | 1.21 (0.71–2.05) | 0.484 |  |  |
| BMI |  |  |  |  |
| ≤ 23.32 | 1.00 |  |  |  |
| > 23.32 | 0.90 (0.54–1.52) | 0.700 |  |  |
| ECOG PS |  |  |  |  |
| 0 | 1.00 |  |  |  |
| ≥ 1 | 1.65 (0.98–2.77) | 0.058 |  |  |
| Smoker |  |  |  |  |
| Yes | 1.45 (0.79–2.66) | 0.226 |  |  |
| No | 1.00 |  |  |  |
| Location of primary tumor |  |  |  |  |
| Left | 1.00 |  |  |  |
| Right | 0.96 (0.54–1.71) | 0.877 |  |  |
| Both | 2.11 (0.29–15.50) | 0.463 |  |  |
| Metastatic organ count |  |  |  |  |
| 1 | 1.00 |  |  |  |
| 2 | 2.44 (1.17–5.10) | 0.018 |  |  |
| 3 | 2.94 (1.38–6.26) | 0.005 |  |  |
| ≥ 4 | 3.66 (1.40–9.57) | 0.008 |  |  |
| Neutrophil-lymphocyte ratio (NLR) |  |  |  |  |
| ≤ 3.2 | 1.00 |  |  |  |
| > 3.2 | 1.33 (0.77–2.29) | 0.305 |  |  |
| Adverse events |  |  |  |  |
| Yes | 0.88 (0.51–1.53) | 0.657 |  |  |
| No | 1.00 |  |  |  |
| Line of immunotherapy |  |  |  |  |
| First-line | 1.00 |  | 1.00 |  |
| Second-or-higher-line | 5.88 (2.11–16.41) | 0.001 | 5.88 (2.11–16.41) | 0.001 |
| Immunotherapy regimen |  |  |  |  |
| Solo therapy | 1.00 |  |  |  |
| Combination therapy | 1.78 (0.43–7.30) | 0.424 |  |  |

Variables are expressed as n (%).

BMI, body mass index; CI, confidence interval; ECOG PS, Eastern Cooperative Oncology Group Performance Status; HR, hazard ratio; mCRC, metastatic colorectal cancer; MSI-L, microsatellite instability-low; MSS, microsatellite stable; TMB, Tumor mutation burden.

**Supplemental Table 8**. Univariable and multivariable Cox regression analyses to identify the risk factors for progression-free survival of the monotherapy subgroup

| Variables | Univariable  HR (95% CI) | *P* value | Multivariable  HR (95% CI) | *P* value |
| --- | --- | --- | --- | --- |
| Age, years |  |  |  |  |
| ≤ 45 | 1.00 |  |  |  |
| > 45 | 1.64 (0.50–5.35) | 0.411 |  |  |
| ECOG PS |  |  |  |  |
| 0 | 1.00 |  | 1.00 |  |
| ≥ 1 | 6.20 (2.02–19.08) | 0.001 | 18.05 (3.86–84.45) | 0.000 |
| Metastatic organ count |  |  |  |  |
| 1 | 1.00 |  | 1.00 |  |
| 2 | 1.12 (0.33–3.86) | 0.855 | 0.41 (0.10–1.62) | 0.202 |
| 3 | 4.59 (1.13–18.66) | 0.033 | 11.62 (2.22–60.98) | 0.004 |
| Microsatellite status |  |  |  |  |
| MSS/MSI-L | 1.00 |  |  |  |
| MSI-H | 0.38 (0.12–1.19) | 0.097 |  |  |
| Tumor mutation burden (Mb) |  |  |  |  |
| < 10 | 2.49 (0.76–8.16) | 0.133 |  |  |
| ≥ 10 | 1.00 |  |  |  |
| Line of immunotherapy |  |  |  |  |
| First-line | 1.00 |  |  |  |
| Second-or-higher-line | 2.93 (0.94–9.17) | 0.065 |  |  |
| Monotherapy regimen |  |  |  |  |
| Nivolumab | 1.00 |  |  |  |
| Pembrolizumab | 0.35 (0.06–1.99) | 0.236 |  |  |
| Toripalimab | 1.46 (0.28–7.69) | 0.653 |  |  |
| Sintilimab | 0.67 (0.11–4.13) | 0.664 |  |  |
| Camrelizumab | 0.00 (0.00– ) | 0.987 |  |  |

Variables are expressed as n (%).

CI, confidence interval; ECOG PS, Eastern Cooperative Oncology Group Performance Status; HR, hazard ratio; mCRC, metastatic colorectal cancer; MSI-L, microsatellite instability-low; MSS, microsatellite stable; TMB, Tumor mutation burden.

**Supplemental Table 9**. Univariable and multivariable Cox regression analyses to identify the risk factors for progression-free survival of the combination therapy subgroup

| Variables | Univariable  HR (95% CI) | *P* value | Multivariable  HR (95% CI) | *P* value |
| --- | --- | --- | --- | --- |
| Age, years |  |  |  |  |
| ≤ 45 | 1.00 |  |  |  |
| > 45 | 1.45 (0.96–2.18) | 0.075 |  |  |
| ECOG PS |  |  |  |  |
| 0 | 1.00 |  |  |  |
| ≥ 1 | 1.31 (0.93–1.84) | 0.124 |  |  |
| Metastatic organ count |  |  |  |  |
| 1 | 1.00 |  | 1.00 |  |
| 2 | 2.00 (1.27–3.13) | 0.003 | 1.26 (0.78–2.04) | 0.337 |
| 3 | 2.92 (1.81–4.70) | 0.000 | 1.98 (1.21–3.26) | 0.007 |
| ≥ 4 | 7.13 (3.82–13.32) | 0.000 | 3.55 (1.85–6.81) | 0.000 |
| Microsatellite status |  |  |  |  |
| MSS/MSI-L | 1.00 |  |  |  |
| MSI-H | 0.22 (0.10–0.51) | 0.000 |  |  |
| Tumor mutation burden (Mb) |  |  |  |  |
| < 10 | 3.00 (1.80–4.99) | 0.000 | 2.44 (1.45–4.13) | 0.001 |
| ≥ 10 | 1.00 |  | 1.00 |  |
| Line of immunotherapy |  |  |  |  |
| First-line | 1.00 |  | 1.00 |  |
| Second-or-higher-line | 3.87 (2.44–6.16) | 0.000 | 3.04 (1.84–5.00) | 0.000 |
| Combination therapy regimen |  |  |  |  |
| Immunotherapy+chemotherapy | 1.00 |  |  |  |
| Immunotherapy+Target therapy | 0.75 (0.32–1.73) | 0.495 |  |  |
| Immunotherapy+Target therapy+chemotherapy | 2.90 (1.85–4.55) | 0.000 |  |  |
| Types of PD-1 inhibitors |  |  |  |  |
| Nivolumab | 1.00 |  |  |  |
| Pembrolizumab | 0.48 (0.18–1.23) | 0.125 |  |  |
| Toripalimab | 1.44 (0.73–2.82) | 0.296 |  |  |
| Sintilimab | 1.20 (0.64–2.73) | 0.570 |  |  |
| Tislelizumab | 0.71 (0.22–2.22) | 0.550 |  |  |
| Camrelizumab | 1.56 (0.57–4.22) | 0.385 |  |  |

Variables are expressed as n (%).

CI, confidence interval; ECOG PS, Eastern Cooperative Oncology Group Performance Status; HR, hazard ratio; mCRC, metastatic colorectal cancer; MSI-L, microsatellite instability-low; MSS, microsatellite stable; TMB, Tumor mutation burden.
